# Editorial: Metabolomic analysis of plant matrices: identification of compounds of therapeutic and nutraceutical interest

**DOI:** 10.3389/fmolb.2026.1996217

**Published:** 2026-09-29

**Authors:** Mariacaterina Lianza, Victor Aguilar-Hernández

**Affiliations:** 1 Division of Pharmacognosy and Natural Products Chemistry, Department of Pharmacy, National and Kapodistrian University of Athens, Athens, Greece; 2 Unidad de Biología Integrativa, Centro de Investigación Científica de Yucatán, Mérida, Yucatán, Mexico

**Keywords:** bioactive compounds, metabolomics, natural products, nutraceuticals, plant metabolites, plant metabolomics, therapeutic compounds

Plants produce an extraordinarily diverse repertoire of metabolites (Pichersky and Lewinsohn, 2011). Their composition changes with tissue, developmental stage, environmental conditions, and processing, providing plants with a remarkable chemical capacity that underlies both their biology and their immense therapeutic and nutraceutical potential. Yet this chemical richness also presents a fundamental analytical challenge. The biological properties of plant matrices rarely reflect the action of a single compound; rather, they emerge from complex mixtures whose constituents vary in identity, abundance, and biological activity.

Metabolomics has provided a powerful approach to explore this complexity by enabling broad interrogation of the chemical space within plant matrices (Shen et al., 2023). In particular, chromatographic separation coupled with high-resolution mass spectrometry, tandem mass spectrometry, and molecular networking can reveal hundreds to thousands of molecular features, providing an increasingly comprehensive view of plant chemical diversity (Wolfender et al., 2013). However, greater analytical coverage does not necessarily translate into greater biological understanding. As detected features multiply, the challenge shifts from data collection to determining which features correspond to genuine metabolites, establishing their structures with confidence, and relating their presence to the properties of the matrix.

Thus, the central challenge is no longer simply detecting more metabolites, but moving from chemical features to reliable metabolite identities and, ultimately, to biological meaning. Achieving this requires rigorous annotation, explicit levels of identification confidence, orthogonal evidence whenever possible to reduce false positives or overcome challenges posed by isomeric forms, and careful integration of chemical and biological information (Schymanski et al., 2014). Metabolomics should be seen not only as a means to increase metabolite coverage, but as a framework for connecting the chemical complexity of plant matrices with compounds and metabolic patterns associated with their biological, nutritional, nutraceutical, and therapeutic properties of plant-derived matrices.

This Research Topic brings together four contributions that examine this challenge across diverse plant systems, pursuing distinct aims ranging from metabolic differentiation and phytochemical characterization to biotechnological production and translational nutraceutical research. Together, they illustrate both the analytical breadth of metabolomics and the challenges of relating chemical patterns to biological meaning.

Chen et al. examine how specialized metabolism varies among wood morphotypes of *Dracaena cochinchinensis* during resin formation. By combining untargeted UPLC-Q-TOF-MS/MS, Feature-Based Molecular Networking (FBMN), multivariate statistics, and pathway enrichment, the authors resolved distinct chemical signatures among three morphotypes and annotated 299 specialized metabolites at MSI Level 2, including flavonoids, phenylpropanoids, steroids, and lipids. The metabolic differences are consistent with a progressive reorganization of specialized metabolism during resin formation, potentially reflecting a transition from structural repair toward chronic adaptation and acute chemical defense. Although this proposed progression remains interpretative rather than causally demonstrated, the study illustrates how metabolomic profiles can reveal coordinated metabolic changes associated with distinct physiological states.

Afzal et al. illustrate how chemical diversity across *Salvia* species can be explored through complementary metabolomic and phytochemical approaches and related to reported pharmacological activities. Their systematic review of 59 species and publications from 2020 to 2025 identified 273 metabolites associated with diverse reported pharmacological activities, with phenolic compounds representing one of the major chemical classes and more than 160 polyphenols reported across the reviewed species. Metabolite abundance varied substantially, with rosmarinic acid among the most prevalent compounds, while essential oil composition likewise differed markedly among species. By integrating evidence from complementary analytical approaches, the review illustrates both the richness of *Salvia* chemistry and the difficulty of relating that diversity to biological activity, as metabolite composition and abundance can vary with species, plant part, extraction conditions, and analytical approach. Thus, although the reported metabolites provide a broad foundation for therapeutic and nutraceutical research, establishing their biological significance will require further mechanistic characterization and compound-level validation.

Laffon et al. examine how metabolomic profiling can inform the optimization of a plant tissue culture system for specialized-metabolite production. Established Populus nigra *in vitro* shoot cultures were grown in Magenta™ vessels and RITA® temporary immersion bioreactors, revealing distinct effects of the culture system and elicitation on growth and phenolic accumulation. The RITA® system increased total phenolic productivity by 160%, with optimal growth and phenolic production achieved after 3 weeks of culture. Methyl jasmonate and salicylic acid further altered the metabolic profile by selectively inducing flavan-3-ols and proanthocyanidins rather than producing a generalized increase in phenolic metabolites. The study also identified 37 metabolites, including 32 reported for the first time in P. nigra. Thus, the culture environment and elicitation regime can redirect specialized metabolism toward defined metabolite classes, providing a basis for optimizing specialized-metabolite production. However, limitations in metabolite identification and the absence of a direct biological activity assessment of RITA®-derived extracts leave the functional significance of these metabolic changes to be established.

Fayomi et al. extend the scope of the Topic beyond plant metabolite characterization by examining whether metabolic responses to dietary anti-inflammatory (poly)phenols remain recognizable across biological systems. Their systematic review of 78 studies spanning *in vitro*, animal, human, and mixed-model investigations found recurring changes in lipid, amino acid, and energy metabolism, whereas SCFAs and urolithins were more closely associated with particular models and sample matrices. Thus, although broad metabolic processes appear repeatedly across experimental systems, the behavior and interpretation of individual metabolites are far less uniform. These findings highlight the potential of metabolomics to identify candidate biomarkers of exposure or biological response, while revealing a fundamental constraint: recurring metabolic themes do not necessarily translate into conserved metabolite responses or clinical benefit.

Taken together, these contributions reveal how metabolomics can illuminate different facets of the chemical complexity of plant matrices—from differences in metabolite composition and quantity to metabolic patterns associated with physiological conditions, specialized metabolite production, and responses to dietary compounds. Yet broader metabolic coverage does not necessarily provide greater biological understanding. Establishing the significance of the metabolites detected requires confidence in their annotation, appropriate consideration of biological context in which they occur, and evidence that links metabolic changes with biological function. Future advances will therefore depend not only on expanding metabolic coverage, but on improving standardization of sample preparation, quality control, data reporting and the structural and functional evidence needed to interpret the chemical diversity revealed by metabolomics.
